# Linking Gastroesophageal Reflux Characteristics to Airway Inflammation: Insights from Bronchoalveolar Lavage Cytology in Severe Preschool Wheeze

**DOI:** 10.3390/life15101561

**Published:** 2025-10-06

**Authors:** Ivan Pavić, Iva Topalušić, Ana Močić Pavić, Roberta Šarkanji Golub, Ozana Hofman Jaeger, Iva Hojsak

**Affiliations:** 1 Department of Pulmonology, Allergology and Immunology, Children’s Hospital Zagreb, Klaićeva 16, 10000 Zagreb, Croatia; iva.topalusic89@gmail.com (I.T.); ozanahj@gmail.com (O.H.J.); 2 School of Medicine, University of Split, Šoltanska 2, 21000 Split, Croatia; 3 Referral Center for Pediatric Gastroenterology and Nutrition, Children’s Hospital Zagreb, Klaićeva 16, 10000 Zagreb, Croatia; amocicpavic@gmail.com (A.M.P.); ivahojsak@gmail.com (I.H.); 4 Department of Cytology, Children’s Hospital Zagreb, Klaićeva 16, 10000 Zagreb, Croatia; roberta.sago@gmail.com; 5 School of Medicine, University of Zagreb, Šalata 3, 10000 Zagreb, Croatia; 6 School of Medicine, University Josip Juraj Strossmayer, Josipa Huttlera 4, 31000 Osijek, Croatia

**Keywords:** gastroesophageal reflux disease, recurrent wheeze, multichannel intraluminal-pH monitoring, bronchoalveolar lavage, lipid-laden macrophages

## Abstract

Background: Gastroesophageal reflux disease (GERD) has been implicated in recurrent wheezing, but mechanisms and diagnostic markers remain debated. Multichannel intraluminal impedance-pH (MII-pH) monitoring improves reflux detection compared to pH-metry, while bronchoalveolar lavage (BAL) cytology may provide evidence of aspiration-related airway inflammation. Objectives: This study aims to examine the relationship between reflux characteristics, BAL cytology and clinical outcomes in preschool children with severe recurrent wheeze. Methods: Preschool-aged children undergoing combined MII-pH and bronchoscopy for severe recurrent wheeze were included. BAL samples were assessed for lipid-laden macrophages (LLM). Associations between reflux parameters, BAL cytology and response to antireflux treatment were analysed. Results: GERD was identified in 70% of participants, with weakly acidic and proximal reflux episodes predominating. Children with GERD exhibited significantly higher percentages of LLM compared with those without GERD (12% vs. 1%, *p* < 0.001). LLM percentage correlated with multiple reflux characteristics, including weakly acidic, liquid and proximal reflux (*p* < 0.047; *p* < 0.047 and *p* < 0.047, respectively), as well as symptom indices (*p* < 0.001). Following antireflux therapy, wheezing episodes were substantially reduced. Conclusions: GERD, particularly weakly acidic and proximal reflux, is associated with airway inflammation and recurrent wheeze in preschool children. BAL LLM percentage may serve as a surrogate marker of reflux-related microaspiration. MII-pH monitoring enhances diagnostic accuracy beyond pH-metry and may help guide targeted antireflux interventions.

## 1. Introduction

Wheezing is a common respiratory symptom in preschool children, often occurring due to various causes such as viral infections, asthma and structural abnormalities of the airways [1]. Among others, gastroesophageal reflux disease (GERD) has been implicated as a possible cause of recurrent or persistent wheezing episodes in this age group [2]. The link between GERD and wheezing is thought to be due to mechanisms such as microaspiration of gastric contents and reflex bronchoconstriction triggered by acid exposure of the esophagus [3].

Conventional diagnostic methods for GERD, such as 24 h esophageal pH monitoring, primarily detect acid reflux episodes but may miss weakly acidic or non-acidic events [4]. This limitation is particularly important in young children, whose frequent feedings can buffer gastric contents, leading to a higher prevalence of non-acid reflux [5]. Multichannel intraluminal impedance pH (MII-pH) monitoring bridges this gap by detecting both acid and non-acid reflux episodes, assessing the physical properties of the refluxate (liquid, gas or mixed) and determining the proximal extent of reflux [6,7]. This comprehensive approach improves the detection of reflux events that could contribute to respiratory symptoms.

Despite the advantages of MII-pH monitoring, the relationship between reflux events detected with this method and inflammation of the lower airways has not yet been sufficiently researched. Bronchoscopy with bronchoalveolar lavage (BAL) allows direct visualization of the airways and sampling for cytological analysis, providing insight into the inflammatory status of the lower airways [8,9,10]. Previous studies have investigated the presence of lipid-laden macrophages (LLM), neutrophils and eosinophils in BAL fluid as potential markers of aspiration and airway inflammation, respectively [11,12,13,14,15]. However, the results were inconsistent and the diagnostic utility of these markers in preschool children with wheezing is still controversial [16].

Given these considerations, our study aims to investigate the correlation between MII-pH monitoring results and bronchoscopy and BAL cytology findings in preschool children with severe recurrent wheezing. By elucidating the relationship between the features of esophageal reflux and lower airway inflammation, we hope to improve diagnostic accuracy and develop targeted therapeutic strategies for this patient group.

## 2. Materials and Methods

### 2.1. Study Design

**Figure 1:**
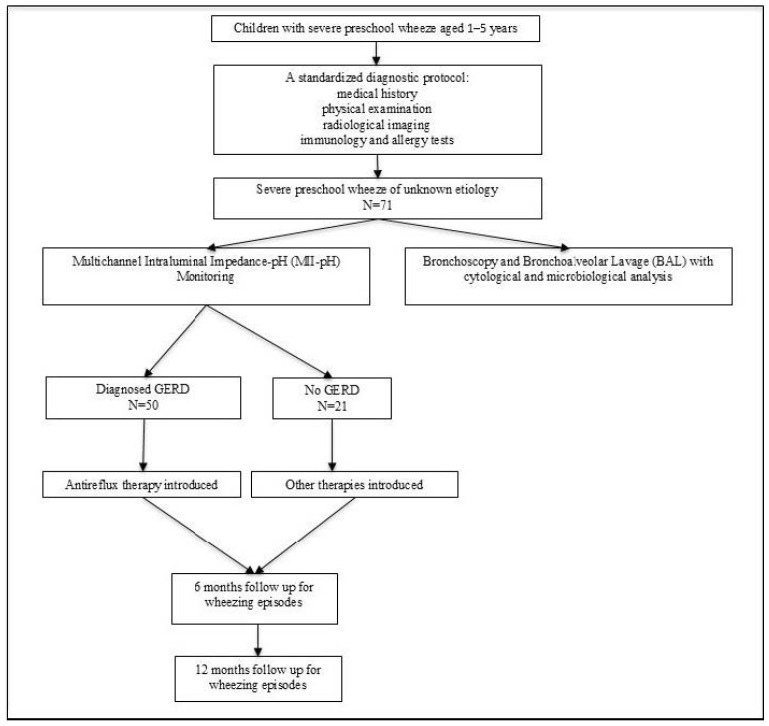
Flowchart of study protocol.

This prospective, single-center, observational study was conducted at the Children’s Hospital Zagreb, a tertiary care centre, between January 2021 and July 2024. Children aged 1 to 5 years, with severe, insufficiently controlled recurrent wheezing despite appropriate medical treatment (ICS and/or montelucast), were consecutively recruited. Eligible participants were referred for both MII-pH monitoring and diagnostic bronchoscopy with BAL (Figure 1).

The inclusion criteria were as follows:

- Age between 12 and 60 months;
- Severe, insufficiently controlled recurrent wheezing (≥4 episodes in 12 months) [17] despite appropriate medical treatment (ICS and/or montelukast);
- No identified underlying etiology after standardized diagnostic work-up.

The exclusion criteria were as follows:

- Congenital or structural abnormalities of the airways;
- Prematurity;
- Neurological impairments affecting swallowing;
- Known primary immunodeficiency;
- Current respiratory infections;
- Suspected or proven asthma;
- Cardiac disease;
- Use of proton pump inhibitors;
- Antibiotic therapy within one month prior to enrolment;
- Patients who experienced wheezing exclusively during viral infections.

Before enrolment in the study, the purpose, procedures and possible consequences were carefully explained and discussed with the parents or carers of each child. Only children who met the predefined inclusion criteria and whose parents or carers provided written informed consent were included in the study. Prior to participation, each child underwent a standardised diagnostic protocol. This included a comprehensive medical history and a detailed physical examination by the same pediatric pulmonologist, as well as appropriate radiological imaging, immunological tests and allergy tests. These investigations were performed to ensure consistency and to exclude other possible underlying conditions. Children with persistent symptoms that could not be explained by other conditions, and in whom routine tests such as medical history, ENT examination, immune and allergy tests, sweat chloride analysis and imaging did not reveal a clear cause, were referred for further investigation. This included bronchoscopy with bronchoalveolar lavage (BAL) and multichannel intraluminal impedance pH (MII-pH) monitoring.

The study was conducted in accordance with the ethical principles of the Declaration of Helsinki and was approved by the Ethics Committee of the Children’s Hospital Zagreb (ID: 02-23/44-2-25).

### 2.2. Multichannel Intraluminal Impedance-pH (MII-pH) Monitoring

All enrolled children underwent 24 h MII-pH monitoring using an age-appropriate impedance pH probe with an ambulatory MII-pH system (Ohmega, MMS, Enschede, The Netherlands).

The MII-pH probe was introduced at the end of the bronchoscopy procedure with BAL. This ensured that MII-pH monitoring and BAL were performed in close temporal proximity, minimizing variability due to changes in clinical status. The probe was inserted nasally, so that the pH electrode was located on the third vertebral body above the diaphragmatic angle, with positioning confirmed radiographically. Parents were trained to keep a careful diary and use an event marker in the data logger to record meals, body position, daily activities and timing of symptoms. The MII-pH recordings were uploaded to a PC at the end of the recording period and manually analysed using the MMS software package by pediatrician who was blinded to the bronchoscopy results. Impedance recordings were analysed using the criteria described in a consensus statement on indications, methodology and interpretation of combined MII-pH monitoring in children [7].

Reflux episodes were defined as follows: liquid reflux was identified by a retrograde drop in impedance to <50% of baseline in at least two consecutive distal impedance channels (≥4 cm or 6 cm above the LES in infants or children, respectively); gas reflux by a rapid increase in impedance >3000 Ω in at least two channels, with at least one >7000 Ω; and mixed reflux as gas occurring during or immediately before liquid reflux. Events reaching the uppermost impedance channels (1 and/or 2) were considered proximal. Reflux was further classified by pH: acid (pH < 4), weakly acidic (pH 4–7), weakly alkaline (pH ≥ 7), and pH-only reflux (pH < 4 for ≥5 s without impedance change). Abnormal acid exposure time (AET), also called reflux index (RI), was defined as >10% in infants and >7% in children over 1 year. An abnormal reflux burden was defined as >100 reflux episodes in infants or >70 in older children. Symptom–reflux correlation was assessed using the Symptom Index (SI ≥ 50%), Symptom Sensitivity Index (SSI ≥ 10%), and Symptom Association Probability (SAP ≥ 95%) [7]. During MII-pH monitoring, parents were asked to record symptoms such as wheezing, coughing, and other respiratory complaints. Among these, wheezing was the primary symptom considered for the calculation of SI, SSI, and SAP, as it represented the main clinical concern in our study population.

### 2.3. Bronchoscopy and Bronchoalveolar Lavage (BAL)

Flexible fiberoptic bronchoscopy was performed under general anaesthesia according to institutional protocols and the guidelines of the European Respiratory Society [18]. Bronchoscopy and BAL were performed by the same interdisciplinary team of pediatric pulmonologists and anaesthesiologists under general anaesthesia (administered by inhalation or intravenously). All procedures were performed in the operating room. BAL was performed using flexible fiberoptic bronchoscopy. Fluid was collected from the right middle lobe or another site selected based on bronchoscopic findings. Sterile saline was instilled in aliquots of 1 mL/kg (maximum 20 mL per aliquot), repeated three times. The BAL fluid was prepared for cytological and microbiological analysis. The first non-centrifuged aliquots were used for microbiological analysis, while subsequent aliquots were processed for cytology.

For cytological analyses, the total cell count was determined using a Fuchs–Rosenthal counting chamber. Smears for differential cell counts (neutrophils, eosinophils, macrophages, lymphocytes) were prepared by cytocentrifugation and stained with May-Grünwald-Giemsa (Polysciences, Inc., Warrington, PA, USA).

Lipid-laden macrophages (LLM) were assessed using Oil Red O staining and quantified as a ratio of LLM to total alveolar macrophages (LLM/AM, %), as previously described [19]. For each specimen, the first 300 AM encountered were examined under light microscopy. Cells containing intracellular Oil Red O-positive lipid droplets were classified as LLM. The percentage of LLM was calculated as the number of LLM divided by the total number of AM counted (300) and expressed as LLM/AM (%). Only samples with adequate cellularity and at least 50% alveolar macrophages were included; those with poor preservation or insufficient cells were excluded. All cytological evaluations were performed by a single experienced cytologist using light microscopy. The cytologist was blinded to clinical and bronchoscopy data, ensuring consistency and reliability of results. Although inter-rater reproducibility was not formally assessed, previous studies have shown that LLM measurements by trained cytologists are reliable and correlate well with established indices such as LLMI [19]. Bronchoscopy and BAL were well tolerated by all participants.

### 2.4. Data Collection and Outcomes

Clinical data, including demographics, history of wheezing, medication use and dietary habits, were collected. The primary outcome was to correlate MII-pH reflux parameters and BAL cytology results. Secondary outcomes included frequency of abnormal reflux events (defined by age-specific thresholds), correlation between reflux type and airway inflammation (neutrophilia, eosinophilia, LLM) and prevalence of abnormal bronchoscopy findings with severe preschool wheeze. Initiation or adjustment of therapy occurred only after both procedures and data collection were completed, ensuring that the measurements reflected the patients’ untreated baseline status. Finally, patients were followed up for 6 and 12 months, after which the outcome of GERD treatment as an add-on therapy of severe preschool wheeze was evaluated. All wheeze episodes were clinician-confirmed, both before enrolment and during the follow-up period. No caregiver diaries or retrospective chart reviews were used to ascertain outcomes; episode counts were based solely on direct clinical assessment and documentation by treating physicians, ensuring accurate and verified outcome reporting.

Statistics. The Kolmogorov–Smirnov test was applied to test the distribution. Differences in the distribution of qualitative variables were determined with the χ^2^ test, while the differences in quantitative variables were determined nonparametric Mann–Whitney U test due to non-normal distribution. Correlation was performed by Pearson correlation. Due to numerous correlations, a Benjamini–Hochberg method was employed to control the false discovery rate. Binary logistic regression was used to assess factors associated with a reduction in wheezing episodes. Statistical analysis was performed using SPSS 25.0 (Chicago, IL, USA) statistical software. The number of obstruction episodes across time was assessed by using generalized linear mixed models (GLMM) with a Poisson distribution and a log link function. To account for within-subject correlation of repeated measures, a random intercept for each subject was included, and the repeated structure was specified with Index1 (three follow-up time points) as the within-subject factor. Receiver operating characteristic (ROC) curve analysis was performed to evaluate the discriminatory ability of the LLM test to distinguish GERD. Statistical significance was set at *p* < 0.05. All analyses were performed in SPSS Statistics version 26.0 (IBM Corp., Armonk, NY, USA).

## 3. Results

The study included 71 children, 49 (69%) boys and 22 (31%) girls, with a mean age of 46.2 (13.1) months. Based on the MII, 50 children (70%) with severe preschool wheeze were diagnosed with GERD, while only 11 children (16%) were diagnosed with GERD by pH-metry. There were no significant differences between the GERD and non-GERD groups in terms of age, body weight, height or BMI Z-scores. The proportion of females was lower in the GERD group than in the non-GERD group (24% vs. 48%, *p* = 0.05) (Table 1).

Children with GERD had a significantly higher number of reflux episodes detected by both pH-metry and impedance than children without GERD. GERD patients had significantly more liquid, mixed, gas, acid, weakly acid and proximal reflux episodes, while non-acid reflux did not differ between groups. The symptom association indices (SI, SSI and SAP) were also significantly higher in the GERD children and bolus clearance time was longer in the GERD group. Data are presented in Table 2.

### 3.1. Association of MII-pH with BAL Cytology, BAL Cultures and Bronchoscopy Findings

The number of BAL cells and the percentage of macrophages, lymphocytes, monocytes, neutrophils, eosinophils and epithelial cells did not differ significantly between children with and without GERD and preschool wheeze (Table 2). However, LLMs were observed more frequently in GERD children (12% vs. 1%, *p* < 0.001) (Table 2). Overall, 38 children (54%) had an LLM percentage of more than 10%, all of whom were in the GERD group (all 38 (76%); *p* < 0.001).

ROC analysis for LLM% that can diagnose GERD showed an area under the curve (AUC) of 0.927 (*p* < 0.001, 95% CI 0.866–0.988) with the best cut-off value of 5.5% of LLM that can predict GERD with a sensitivity of 86% and specificity of 100%.

A total of 12 children with recurrent wheezing had positive BAL cultures. The most frequently isolated organisms were *Haemophilus* (*H*.) *influenzae* (*n* = 6), *Moraxella* (*M*.) *catarrhalis* (*n* = 4) and *Streptococcus* (*Str*.) *pneumoniae* (*n* = 2). Positive cultures were significantly more common in the non-GERD group than in the GERD group [5/15 (33%) vs. 7/67 (10%), *p* = 0.017] (Table 2).

The presence of macroscopic bronchoscopic changes did not differ significantly between preschool wheeze patients with and without GERD (Table 2).

### 3.2. Correlation of Reflux Parameters and BAL Cytology Findings

Correlation analysis showed that the percentage of LLM was significantly associated with the number of total, acidic, weakly acidic, liquid and mixed reflux episodes and with SI (Table 3). No correlations were found between the reflux parameters and the total number of BAL cells, the macrophage percentage or the neutrophil percentage (Table 3).

In univariate logistic regression, neither GERD diagnosis, gender, age nor specific therapeutic interventions were significantly associated with the absence of wheezing episodes 6 months after MII-pH (Table 4). Antireflux therapy and dietary modifications showed a borderline association (OR = 3.548, 95% CI 0.916–13.724, *p* = 0.067).

### 3.3. Clinical Outcomes of GERD-Related Therapeutic Interventions in Children with Preschool Wheeze During 6- and 12-Month Follow-Up

At both the 6- and 12-month follow-up, the number of wheezing episodes was significantly reduced in both the GERD and non-GERD groups compared to the period before the MII-pH monitoring (all *p* < 0.001).

**Figure 2:**
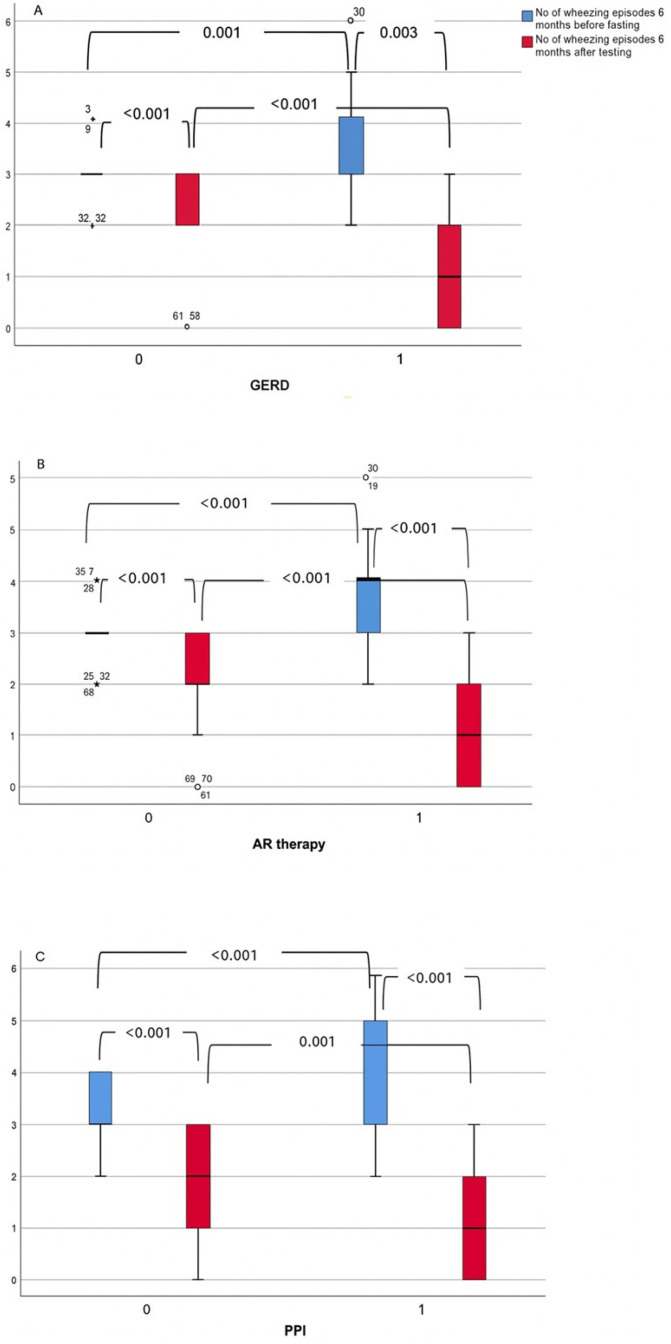
(**A**–**C**) The number of wheezing episodes at 6-month follow-up. 0—No; 1—Yes; *p* values showing difference between measurements are presented in the figures. Boxplots display medians and interquartile ranges; whiskers cover most of the remaining data and any data points beyond.

**Figure 3:**
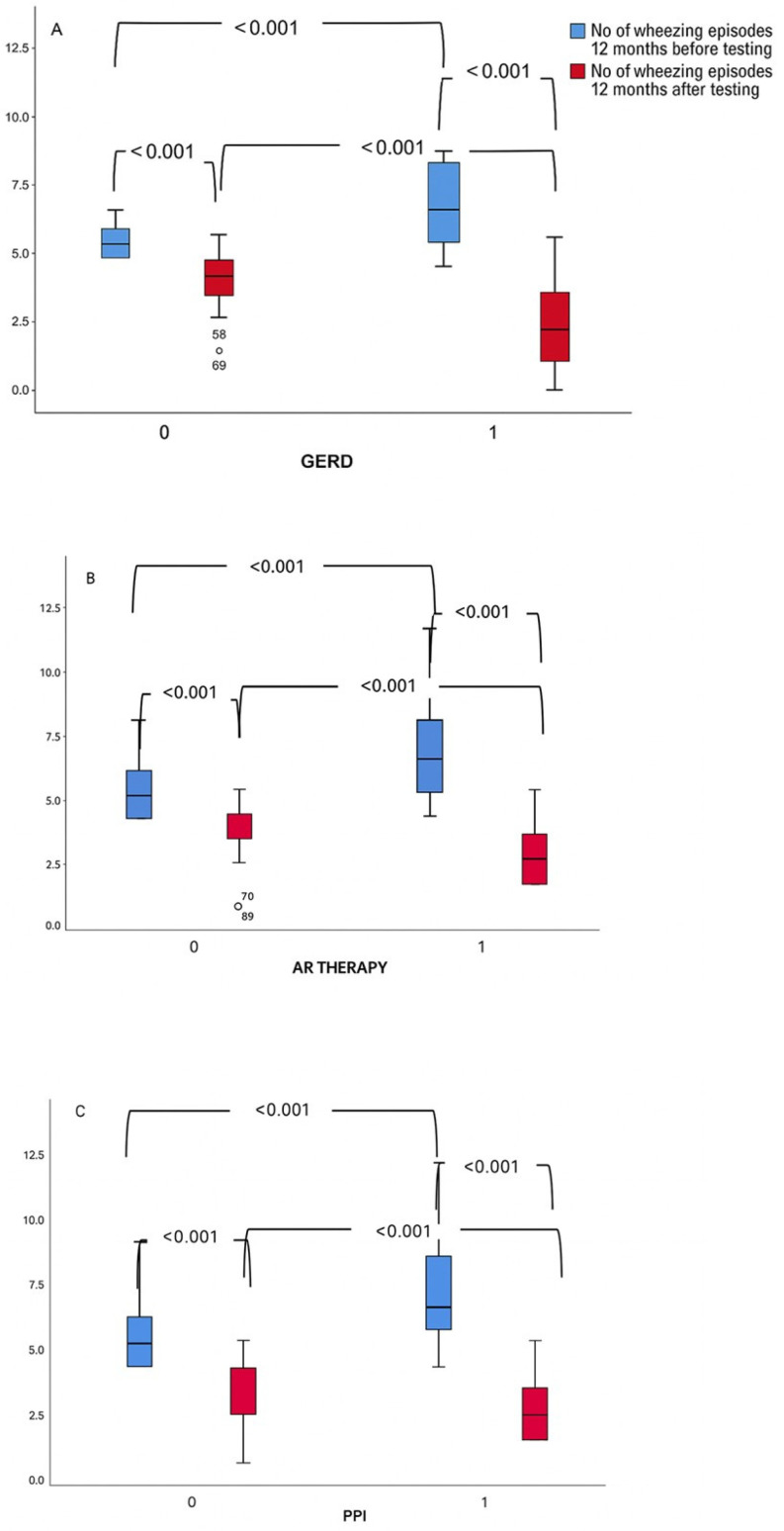
(**A**–**C**) The number of wheezing episodes at 12-month follow-up. 0—No; 1—Yes; *p* values showing difference between measurements are presented in the figures.

Six and 12 months prior to MII-pH, children with GERD had more episodes of wheezing than children without GERD (Figure 2A and Figure 3A). After they had undergone clinical evaluation, MII-pH monitoring and bronchoscopy, most children with GERD received antireflux therapy (90%), including lifestyle changes (90%), PPI (80%) and alginate (28%).

When stratified by antireflux therapy, treated children also had a higher frequency of wheezing episodes before testing, but showed a significant decrease after therapy at both 6 and 12 months (Figure 2B and Figure 3B). A similar trend was observed in children treated with proton pump inhibitors (PPI). They had significantly more wheezing episodes before the test, but these significantly decreased after treatment (Figure 2C and Figure 3C).

GLMM with a Poisson distribution revealed that the main effect of GER status was not statistically significant (IRR = 1.01, 95% CI: 0.90–1.13, *p* = 0.83), indicating no overall difference in obstruction rates between children with normal and abnormal GER as defined by impedance testing. Time had a significant effect on obstruction counts. Compared with the third time point (12 months after diagnosis as reference), the rate of obstruction episodes was 36% higher at baseline (IRR = 1.36, 95% CI: 1.20–1.55, *p* < 0.001) and 48% lower at the second time point (IRR = 0.52, 95% CI: 0.42–0.65, *p* < 0.001). The GER × time interaction was not statistically significant (all *p* > 0.05), suggesting that the time-related pattern of obstruction rates was similar in children with and without abnormal GER.

## 4. Discussion

This study investigated the relationship between GER characteristics assessed by MII-pH monitoring, bronchoscopy with BAL cytology, and clinical outcomes in preschool children with recurrent wheezing. Our results indicate a high prevalence of GERD (70%) diagnosed by MII-pH, with significant differences in BAL cytology, particularly LLM, between children with and without GERD. Notably, a significant decrease in wheezing episodes was observed after initiation of antireflux therapy, suggesting a clinically relevant association between reflux and respiratory symptoms.

The increased detection of GERD by MII-pH monitoring in our cohort is consistent with the findings of Abdullah et al., who reported abnormal MII-pH findings in 60.5% of wheezy infants compared to only 7.9% by pH-metry [20]. Similar to our results, their study showed significantly higher LLM indices in MII-positive infants, emphasizing the potential role of microaspiration in lower airway inflammation. However, their population consisted of infants under one year of age, whereas our cohort included preschool-aged children (mean age 46.2 months). Age-related differences in reflux clearance and airway defense mechanisms could account for differences in severity and presentation. In another study of the same group, pathological reflux by MII-pH was found in over 60% of wheezing infants [21], a prevalence very similar to that observed in our cohort. In addition, they analyzed pepsin from bronchoalveolar lavage as a biomarker for GERD and showed significantly higher pepsin levels in MII-positive infants compared to controls. Although pepsin was not investigated in our study, we observed increased LLM in GERD-positive children and a subsequent reduction in wheezing after antireflux therapy, indirectly supporting the pathogenic role of reflux microaspiration.

In contrast, a prospective study from Boston that examined children undergoing combined MII-pH monitoring and BAL sampling found no significant correlation between LLMI and reflux parameters, suggesting that LLMI may not be a reliable marker of microaspiration across all pediatric populations [22]. Several factors may account for the discrepancy between their findings and ours, including differences in LLMI scoring methods, reflux episode classification, sample size, and timing between MII-pH testing and bronchoscopy. Another important difference lies in the study populations. Our cohort was limited to preschool-aged children with severe recurrent wheeze (mean age 46.2 months) and thus represented a relatively homogeneous group, both in terms of age and clinical presentation. In contrast, the Boston study included a broader and more heterogeneous population (mean age 6.4 ± 5.2 years) with different indications for bronchoscopy and MII-pH testing. The broader case mix in their study may have diluted the potential association between reflux and LLMI, while the more uniform clinical profile in our cohort may have allowed clearer detection of this relationship.

Further evidence supports a pathogenic role of GER in wheezing children. Sheikh et al. showed that 64% of infants with wheezing had abnormal pH studies, and of these, almost two-thirds were able to discontinue daily asthma medications after antireflux treatment, whereas this was not the case in the GER-negative group [23]. This emphasizes that treatment of reflux can directly improve respiratory outcomes, even when reflux is clinically silent. Similarly, Saglani et al. found that two-thirds of children with severe recurrent wheeze had abnormal reflux studies, in addition to various airway abnormalities on bronchoscopy and BAL, such as mucus plugging, inflammation, and eosinophilia [24]. While their study highlighted the complexity of airway pathology in young children, our results show that reflux identified by MII-pH monitoring is associated with increased LLM indices and that antireflux therapy can reduce wheezing. Taken together, these data support the view that reflux is not merely an associated finding but may be an important and treatable contributor to respiratory disease in early childhood. In our recent prospective study, we found a comparable prevalence of GERD (71%) based on MII-pH [25]. A significant reduction in wheezing episodes following antireflux therapy was reported, mirroring the present results. However, statistically significant differences in BAL cytology between GERD and non-GERD groups, including LLM, were not found. This discrepancy could be explained by the smaller sample size in non-GERD, which limits statistical power.

Although most reflux events in our cohort were non-acidic or weakly acidic, PPI therapy was still used. This approach aligns with current guideline-based practice, in which PPIs remain the first-line therapy for GERD regardless of reflux type [26]. Many weakly acidic reflux events originate as acid reflux that is subsequently partially neutralised by saliva or ingested food; PPIs reduce the initial acid component and thus decrease the risk of mucosal irritation. In addition, PPIs lower overall gastric secretions, including pepsin activity and bile salt solubility, which may still contribute to airway inflammation [27,28]. Moreover, PPIs serve a dual purpose in this context: providing potential symptom relief and functioning as a therapeutic trial to identify patients who may require further diagnostic evaluation. Thus, while our findings highlight a predominance of non-acid reflux, the use of PPIs remains clinically justified as an initial therapeutic approach.

Several studies have questioned the specificity of LLM as a marker for aspiration and have pointed out that elevated LLM can also occur in various lung diseases [11,16,29]. A study published by Kazachkov et al. found that children with chronic respiratory symptoms had higher LLMI than adult controls, but no consistent correlation with reflux parameters was found [30]. These results suggest that LLMs may result from various airway insults, and not exclusively from microaspiration.

In our study, although the percentage of LLM in BAL was relatively low (12%), it was still significantly higher in GERD-positive than in GERD-negative children. This modest prevalence inherently limits both the sensitivity and negative predictive value of BAL as a diagnostic tool for reflux-related microaspiration. Therefore, while LLM may provide supportive evidence of aspiration in GERD, it should not be considered a standalone diagnostic marker. Instead, BAL findings should be interpreted alongside other modalities, such as MII–pH monitoring and clinical symptom indices, as part of a multimodal diagnostic approach. Additionally, we performed an ROC analysis to further evaluate the diagnostic performance of LLM percentages. The results showed that LLM percentages could predict GERD with an area under the curve (AUC) of 0.927 (*p* < 0.001, 95% CI 0.866–0.988). A threshold value of >5.5% LLM yielded a sensitivity of 86% and a specificity of 100% for diagnosing GERD. These findings indicate that, despite the relatively low prevalence of LLM in BAL, even modest increases in LLM percentages may have substantial diagnostic value in identifying reflux-related disease.

Nevertheless, LLM correlated significantly with reflux parameters such as weakly acidic, mixed, liquid and proximal reflux episodes, as well as with symptom indices. These associations strengthen the hypothesis that aspiration of refluxate plays a pathophysiological role in this subgroup of preschool children. In addition, our analysis revealed that weakly acidic and mixed reflux episodes, which are often not detected by pH monitoring alone, were significantly associated with increased LLM percentages. This emphasizes the importance of impedance measurements in the assessment of non-acidic reflux, particularly in children, where feeding patterns and buffering effects make acid reflux difficult to detect [31].

Interestingly, other inflammatory markers, such as neutrophils, lymphocytes, eosinophils, and total cell count in the BAL, did not differ significantly between the groups. This suggests that airway inflammation in GERD-related wheezing may be subtle and specific to microaspiration rather than overt infectious or eosinophilic inflammation. Of note, the absence of eosinophilic inflammation in our cohort may be partially explained by prior treatment with inhaled corticosteroids (ICS) [32]. It is possible that children with eosinophilic airway inflammation, who are known to respond well to ICS, needed no further diagnostic investigations, including MII-pH, bronchoscopy, and BAL. Consequently, this group of responders may be underrepresented in our study population, which may explain the lack of eosinophilic inflammation observed.

Consistently, bacterial positivity in BAL was more common in non-GERD patients (33% vs. 10%, *p* = 0.017), possibly indicating alternative etiologies such as infection-related wheezing in this subgroup. Our microbiological analysis revealed positive BAL cultures in 12 children, with *H*. *influenzae*, *M*. *catarrhalis*, and *S*. *pneumoniae* being the most frequently isolated organisms. These pathogens are consistent with previous reports of wheezing children, which, according to some studies, reach a prevalence of 50% [33,34,35,36,37]. For example, De Schutter and colleagues identified the same bacterial species but reported a higher overall rate of positive BAL cultures, possibly due to differences in study populations, sampling methods, or local epidemiological factors influencing bacterial colonization [38]. In particular, neonatal cohort studies, such as the COPSAC 2000 study, have shown that early colonization with *H*. *influenzae*, *M*. *catarrhalis*, and *S*. *pneumoniae* is associated with an increased risk of persistent wheezing and asthma in the first five years of life [39]. Robinson et al. also identified a cluster with high prevalence of infection as one of four clusters of severe preschool wheeze [13]. Interestingly, the higher culture positivity in the non-GERD group in our cohort suggests that bacterial colonization preferentially contributes to wheezing in children without reflux. This supports the concept that recurrent wheezing may result from different pathogenic mechanisms: microaspiration and reflux-related airway inflammation in GERD-positive children vs. bacterial colonization and infection in non-reflux-related wheezing. Taken together, these findings emphasize the importance of considering both infectious and reflux-related mechanisms in the assessment of recurrent wheezing in preschool children.

We found no significant difference in macroscopic bronchoscopy findings between the GERD and non-GERD groups, despite the differences in BAL cytology. This suggests that bronchoscopy can detect structural abnormalities and gross inflammation, but may miss subtle inflammatory processes such as those caused by reflux-induced microaspiration. Therefore, in children with recurrent wheezing and normal bronchoscopy, a pathological BAL or abnormal MII-pH may still reveal clinically significant disease.

Importantly, in children diagnosed with GERD, the number of wheezing episodes decreased significantly more than in non-GERD patients 6 and 12 months after diagnosis. This reduction was associated with a high rate of antireflux therapies, including PPIs, alginates, and diet/lifestyle interventions. These results support the therapeutic benefit of targeted GERD treatment in reducing respiratory morbidity in children with wheezing. Interestingly, children without GERD also showed a statistically significant decrease in wheezing episodes over time. This improvement likely reflects the implementation of other therapeutic strategies tailored to their individual clinical profile. In the group without GERD, treatment may have included inhaled corticosteroids, bronchodilators, antibiotics (particularly in cases with positive BAL cultures), or allergen and environmental control measures. These findings highlight that while GERD-targeted therapy is effective for those with reflux-related symptoms, wheezing in preschool children is multifactorial and requires a comprehensive, individualized treatment approach [40,41]. Although logistic regression did not identify GERD diagnosis or antireflux therapy as statistically significant independent predictors of wheeze resolution (likely due to limited sample size and wide confidence intervals), the observed clinical trend suggests a potential benefit of treating GERD when present. At the same time, the improvement observed in non-GERD patients emphasizes the value of targeted non-GERD therapies for other phenotypes of wheezing.

However, it is important to note several limitations of our study, including relatively small sample size, possible selection bias (children already referred for MII-pH and bronchoscopy) and the lack of a healthy control group. A potential limitation of our study is the selection of participants, as all children were referred for both MII-pH monitoring and bronchoscopy due to severe, treatment-refractory recurrent wheezing. This referral pattern may introduce selection bias, limiting the applicability of our findings to the broader population of children with recurrent wheeze. Therefore, our results are most relevant to high-risk pediatric patients with severe or poorly controlled symptoms, and caution should be used when extrapolating these findings to children with milder disease or those with an identifiable underlying etiology. Future studies including a more representative spectrum of wheezing children would help to validate and extend these findings. In addition, although we quantified the percentage of macrophage populations in the BAL, absolute LLMI was not formally assessed, which may limit direct comparability with other studies. This approach is supported by previous studies. Kitz et al. demonstrated a strong correlation between LLMI and percentage LLM quantification (Pearson’s r = 0.9890), suggesting that the latter may be a valid and more practical surrogate [19]. In addition, Ezmigna and colleagues focused on the percentage of LLM, rather than the traditional LLMI scoring system, in their study of bronchoalveolar lavage profiles in children with uncontrolled wheeze [42]. They used a threshold of ≥20% LLM, noting this to be roughly equivalent to an LLMI of 80–100, but did not formally score LLMI. Although they did not directly compare LLMI and LLM percentages, their method implies a pragmatic equivalence. This supports the idea that percentage LLM can serve as a reasonable, simplified substitute for LLMI. Another potential limitation is recall bias in reporting pre-test wheezing episodes, which may have affected the accuracy of symptom counts. Misclassification related to prior ICS use is also possible, as ICS may suppress eosinophilic inflammation and alter BAL cytology, potentially obscuring the underlying airway phenotype. Furthermore, preschool wheeze represents a heterogeneous condition with overlapping endotypes, including reflux-predominant, infection-predominant, and atopy-related phenotypes. Our analysis did not stratify children according to these phenotypic subgroups, which may have limited the precision of associations between reflux, BAL findings, and clinical outcomes. Stratified analyses or data-driven clustering approaches in larger cohorts would help to better disentangle these mechanisms and refine future endotyping strategies.

## 5. Conclusions

In conclusion, our study provides evidence supporting the role of GER, particularly weakly acidic and proximal reflux, in lower airway inflammation driven by LLM, and recurrent wheezing in preschool children. The association between reflux parameters with LLM, as well as the association of LLM with the symptom indices, suggests that microaspiration is a plausible mechanism in severe preschool wheeze. These findings emphasize the importance of considering reflux as a potential contributor to respiratory symptoms and support the integration of MII-pH monitoring and BAL cytology in the diagnostic work-up of difficult-to-treat preschool wheezing. While antireflux therapy may improve symptoms, a personalized and multidisciplinary approach is essential. Future multicenter studies with standardized BAL cytology scoring and larger cohorts are warranted to validate these findings and clarify the pathophysiological pathways linking reflux and respiratory symptoms. Bacterial infection was another important pathogenic mechanism in difficult-to-control wheeze in preschool children. These findings could contribute to the further endotyping of preschool wheeze, which is still an unmet need in pediatric pulmonology. In addition, phenotypic heterogeneity remains a key challenge in this age group. Future studies using stratified analyses (e.g., infection-predominant vs. reflux-predominant) or unbiased, data-driven clustering approaches may help disentangle overlapping mechanisms and guide more precise endotyping of preschool wheeze.

## Figures and Tables

**Table 1:** Difference in demographic data between patients with gastroesophageal reflux disease vs. those without gastroesophageal reflux disease based on multichannel intraluminal impedance.

	GERD (*n* = 50)	No GERD (*n* = 21)	*p*
Age, months (median, range)	44 (24–60)	54 (28–60)	0.338
Sex (female, %)	12 (24%)	10 (48%)	0.05
Body weight for age Z score (median, range)	0.95 (−3.1 to 3.5)	0.82 (−1.5 to 2.4)	0.811
Body height for age Z score (median, range)	2.15 (−2.1 to 3.9)	1.5 (−0.2 to 4.8)	0.290
BMI for age Z score (median, range)	−0.3 (−3.3 to 2.8)	0.15 (−2 to 1.6)	0.634

**Table 2:** Difference between children with proven gastroesophageal reflux disease based on multichannel intraluminal impedance and children without gastroesophageal reflux disease; Continuous data presented as median (range).

	GERD (*n* = 50)	No GERD (*n* = 21)	*p*
Number of reflux episodes on pH-metry (median, range)	44.5 (1–158)	28 (6–43)	<0.001
Reflux index (median, range)	2.4 (0.1–24.9)	1.8 (0.2–2)	0.094
Number of reflux episodes on MII (median, range)	130.5 (61–336)	48 (19–91)	<0.001
Liquid reflux episodes (median, range)	30.5 (1–86)	15 (0–30)	0.001
Mixed reflux episodes (median, range)	43 (1–110)	15 (2–29)	<0.001
Gas reflux episodes (median, range)	45 (9–231)	10 (0–77)	<0.001
Acid reflux episodes on MII (median, range)	30.5 (9–125)	14 (1–20)	<0.001
Weakly acid reflux episodes on MII (median, range)	84 (27–241)	27 (10–59)	<0.001
Non-Acid reflux episodes on MII (median, range)	5.5 (0–42)	5 (0–34)	0.985
Proximal reflux episodes on MII (median, range)	49 (16–152)	15 (0–19)	<0.001
Bolus CT (median, range)	6.8 (4.3–19.2)	5.7 (3.4–15.9)	0.023
SI (median, range)	54.8 (32–100)	24.9 (5.6–42.8)	<0.001
SSI (median, range)	8.9 (2.1–38.4)	3.4 (1.2–9.8)	<0.001
SAP (median, range)	99.9 (87.6–100)	67.8 (0–89.5)	<0.001
Antireflux therapy, *n* (%)	45 (90%)	1 (5%)	<0.001
Alginate, *n* (%)	14 (28%)	0	0.007
PPI, *n* (%)	40 (80%)	0	<0.001
Lifestyle changes, *n* (%)	45 (90%)	1 (5%)	<0.001
Number of wheezing episodes 6 months before MII-pH, episodes per period (median, range)	4 (2–6)	3 (2–4)	<0.001
Number of wheezing episodes 12 months before MII-pH, episodes per period (median, range)	6 (4–14)	5 (4–8)	<0.001
Number of wheezing episodes in the next 6 months, episodes per period (median, range)	1 (0–3)	3 (0–3)	0.001
Number of wheezing episodes in the next 12 months, episodes per period (median, range)	2 (0–5)	4 (0–5)	<0.001
Number of cells, cells/uL (median, range)	117.5 (34–4056)	113 (71–5065)	0.251
Macrophages (%) (median, range)	90.5 (13–99)	90 (45–98)	0.791
Lipidophages (%) (median, range)	12 (1–41)	1 (0–4)	<0.001
Lymphocytes (%) (median, range)	3 (0–9)	3 (0–5)	0.34
Monocytes (%) (median, range)	0 (0–5)	1 (0–3)	0.222
Neutrophils (%) (median, range)	4 (0–82)	3 (1–50)	0.625
Epithelial cells (%)	0	0	1.0
Eosinophils (%) (median, range)	0 (0–5)	0 (0–6)	0.267
Mast cells (%)	0	0	1.0
BAL—bacterial positive, *n* (%)	5 (10%)	7 (33%)	0.017
Bronchoscopy macroscopic changes, *n* (%)	31 (62%)	10 (48%)	0.263

**Table 3:** Correlation analysis of reflux parameters and bronchoalveolar lavage cytology findings. Due to numerous correlations, a Benjamini–Hochberg method was employed to control the false discovery rate. Adjusted *p* values are listed below *p* values and stated in bold, presented only for statistically significant *p* values.

	Number of Cells in BAL Coefficient	Number of Cells in BAL *p* Value	Macrophage Percentage in BAL Coefficient	Macrophage Percentage in BAL *p* Value	LLM/AM Percentage in BAL Coefficient	LLM/AM Percentage in BAL *p* Value	Neutrophil Percentage in BAL Coefficient	Neutrophil Percentage in BAL *p* Value
Number of reflux episodes, pH-metry	0.018	0.882	−0.137	0.255	0.178	0.137	0.113	0.349
RI, %	0.018	0.503	−0.167	0.164	0.091	0.451	0.152	0.204
Number of reflux episodes, impedance	−0.54	0.657	−0.004	0.974	0.541	<0.001 **0.047**	−0.14	0.906
Number of acidic GER, impedance	−0.073	0.546	−0.099	0.41	0.401	0.001 **0.047**	0.059	0.627
Number of weakly acidic GER, impedance	−0.025	0.836	0.028	0.818	0.448	<0.001 **0.047**	−0.031	0.797
Number of non-acidic GER, impedance	−0.036	0.768	0.079	0.514	−0.009	0.942	−0.081	0.504
Number of liquid GER, impedance	−0.043	0.722	−0.153	0.261	0.498	<0.001 **0.047**	0.113	0.348
Number of mixed GER, impedance	−0.119	0.324	0.073	0.543	0.459	<0.001 **0.047**	−0.064	0.595
Number of gas GER, impedance	−0.025	0.839	0.009	0.943	0.268	0.024 **0.376**	−0.029	0.812
SI, %	−0.095	0.433	0.049	0.685	0.585	<0.001 **0.047**	−0.041	0.735
SSI, %	−0.082	0.499	0.01	0.99	0.115	0.341	−0.20	0.87
SAP, %	−0.014	0.91	0.013	0.918	0.115	0.341	−0.007	0.939

**Table 4:** Binary logistic regression (univariate) showing risk factors for not having wheezing episodes 6 months after MII-pH.

	OR	95% CI	*p*
GERD based on impedance	2.571	0.658–10.056	0.175
Sex	0.865	0.276–2.71	0.803
Age	1.019	0.988–1.051	0.225
Antireflux therapy	3.548	0.916–13.724	0.067
Alginate	0.532	0.151–1.868	0.325
PPI	0.56	0.183–1.714	0.31
Dietary modifications	3.548	0.916–13.725	0.067

## Data Availability

The original contributions presented in this study are included in the article. Further inquiries can be directed to the corresponding author.

## Abbreviations

AM, alveolar macrophage; BAL, bronchoalveolar lavage; BMI, body mass index; GERD, gastroesophageal reflux disease; ICS, inhaled corticosteroid; LLM, lipid-laden macrophage; LLMI, lipid-laden macrophages index; MII-pH, multichannel intraluminal impedance pH; ORO, Oil Red O; PPI, proton pump inhibitor; SI, symptom index; SSI, symptom sensitivity index; SAP, symptom association probability.
